# Long-Term Patient Satisfaction and Quality of Life After Breast-Conserving Therapy: A Prospective Study Using the BREAST-Q

**DOI:** 10.1245/s10434-021-10377-4

**Published:** 2021-07-19

**Authors:** Ilona Stolpner, Jörg Heil, Fabian Riedel, Markus Wallwiener, Benedikt Schäfgen, Manuel Feißt, Michael Golatta, André Hennigs

**Affiliations:** 1 Department of Gynecology and Obstetrics, University of Heidelberg, Heidelberg, Germany; 2 Institute of Medical Biometry and Informatics, University of Heidelberg, Heidelberg, Germany

## Abstract

**Background:**

Poor patient-reported satisfaction after breast-conserving therapy (BCT) has been associated with impaired health-related quality of life (HRQOL) and subsequent depression in retrospective analysis. This prospective cohort study aimed to assess the HRQOL of patients who have undergone BCT using the BREAST-Q, and to identify clinical risk factors for lower patient satisfaction.

**Methods:**

Patients with primary breast cancer undergoing BCT were asked to complete the BREAST-Q preoperatively (T1) for baseline evaluation, then 3 to 4 weeks postoperatively (T2), and finally 1 year after surgery (T3). Clinicopathologic data were extracted from the patients’ charts. Repeated measures analysis of variance (ANOVA) was used to determine significant differences in mean satisfaction and well-being levels among the test intervals. Multiple linear regression was used to evaluate risk factors for lower satisfaction.

**Results:**

The study enrolled 250 patients. The lowest baseline BREAST-Q score was reported for “satisfaction with breast” (mean, 61 ± 19), but this increased postoperatively (mean, 66 ± 18) and was maintained at the 1 year follow-up evaluation (mean, 67 ± 21). “Physical well-being” decreased from T1 (mean, 82 ± 17) to T2 (mean, 28 ± 13) and did not recover much by T3 (mean, 33 ± 13), being the lowest BREAST-Q score postoperatively and in the 1-year follow-up evaluation. In multiple regression, baseline psychosocial well-being, body mass index (BMI), and type of incision were risk factors for lower “satisfaction with breasts.”

**Conclusion:**

Both the aesthetic/surgery-related and psychological aspects are equally important with regard to “satisfaction with breasts” after BCT. The data could serve as the benchmark for future studies.

Breast cancer is the most common malignancy in women. Due to ongoing improvements of treatment methods for early-stage breast cancer, the rate of breast cancer-specific survival has never been higher than it is currently.^1^ With this high portion of women surviving breast cancer beyond treatment, their long-term health-related quality of life (HRQOL) has become an additional focus of outcome research.^2^ Studies have identified HRQOL as an important end point that provides prognostic information for medical outcomes and survival.^3^,^4^ Thus, patient-reported outcome measures (PROM) have found their way into clinical trials on a more regular basis.^5^

The factors of HRQOL are physical functioning, psychological well-being, and social integration. In breast cancer, findings have shown aesthetic outcome to be an additional factor influencing HRQOL.^6^,^7^ The assessment of HRQOL has become an important component of public health surveillance, and self-assessed health status also can be a more powerful predictor of mortality and morbidity than many objective measures of health.^8^,^9^

Usually, HRQOL is assessed from the perspective of the individual by a questionnaire. This subjective evaluation is sometimes viewed skeptically because the use of objective measurement parameters as an end point in clinical studies has a long tradition. However, subjectivity should not a priori be classified as unreliable. Symptoms reported by patients are reproducible with a very high level of validity.^10^

Several studies have been conducted to discover factors predicting the HRQOL of breast cancer patients.^11^–^13^ The common aim is to find possible controllable factors in order to develop targeted interventions. Influencing variables are divided into factors related to the patient, the tumor, or the treatment. General cancer-related factors, such as pain and fatigue, have been associated with worse HRQOL for patients with metastatic cancer.^14^ The patient-related factor of financial difficulties also has been associated with low HRQOL outcomes.^15^ Other factors concerning the patient’s lifestyle (e.g., health literacy, access to health information, and self-efficacy in following a healthy diet or exercise) are reported as contributing to HRQOL.^16^,^17^

Whereas some studies seem to provide similar findings, other studies differ substantially in their conclusions. For example, treatment-related factors, such as the type of incision or postoperative complications, were shown to be risk factors for poor aesthetic outcomes in a prospective cohort study using the Breast Cancer Treatment Outcome Scale (BCTOS) questionnaire,^18^ whereas similar treatment factors were not predictive of the long‐term quality of life (QoL) in another PROM.^13^

Inconsistencies such as these show up throughout the outcome research conducted to date. This may be due to the lack of standardization in the PROMs used in cancer research. The variability of instruments makes it difficult to compare the different studies and available data.^19^

The BREAST-Q questionnaire is a PROM developed especially for breast cancer patients undergoing breast surgery. Independent modules are available for the different surgical interventions (e.g., mastectomy, reconstruction, augmentation). The BREAST-Q questionnaire was developed using extensive patient input and Rasch psychometric methods.^20^,^21^ It is a psychometrically validated and reliable PROM,^22^ serving as a perfect candidate to fill the gap of a standardized measurement instrument. The breast-conserving therapy (BCT) module is the most recently added module, meeting the needs of this patient group.^22^,^23^

Besides the variability of the measurement instruments used, the design of most studies investigating HRQOL after breast cancer surgery might be another disadvantage. Whereas many factors associated with HRQOL have been identified in retrospective cross-sectional surveys, prospective studies with defined time frames including preoperative data at baseline still are rare. Besides, it needs to be emphasized that accurate predictions cannot be guaranteed by cross-sectional studies. Rather, the development of prediction models generally is based on cohort studies.^24^

The BREAST-Q is the best PROM for this study design because each module consists of a preoperative and a postoperative questionnaire.^25^ Although a number of studies have evaluated the BREAST-Q in recent years, most studies using the BREAST-Q for BCT have been retrospective, cross-sectional evaluations that have not used the advantage of baseline data acquisition.^26^–^30^

In this article, we report the BREAST-Q scores for a prospective cohort of patients using the BCT module with preoperative baseline evaluation and two postoperative follow-up assessments. We analyze patient, tumor, and surgical factors predicting HRQOL.

## Methods

### Ethics

The Ethics Commission of the University of Heidelberg Medical School approved the study in August 2017. The study was deemed to be without risk, including only anonymized analysis of routinely collected data. Consequently, the Ethics Committee of the University of Heidelberg did not request consent for this analysis.

### Study Design

This study was designed as an exploratory, single-institution, prospective cohort study.

### Study Sample

Patients were eligible for inclusion in this study if they had primary breast cancer confirmed histologically and were scheduled for BCT between July 2017 and May 2018. Patients were excluded if they opted for mastectomy, had a diagnosis of recurrence or metastasis, or declined to participate in the study.

The patients were informed about the study 1 day to 1 week before the surgery (T1). If they agreed to participate, they completed the BREAST-Q preoperative BCT module. Then, 1 week after their surgery, the patients were sent the BREAST-Q postoperative BCT module (all scales except “side effects of radiation”), together with a letter asking them to complete the questionnaire before the beginning of their radiation treatment (T2). In this way, we avoided assessing the impact of radiotherapy side effects on the HRQOL of patients at T2.

At 1 year after their surgery, the patients were sent the BREAST-Q postoperative BCT module (T3) with a letter asking them to participate in the long-term follow-up evaluation. The scales concerning the experience of care that measure satisfaction with the information provided as well as satisfaction with the surgeon, medical team, and office staff were assessed only once after the surgery (T2). The patients were reminded up to three times by phone to participate in the follow-up assessment. Relevant therapy-related data were extracted from the patients’ charts.

### Surgical Techniques Applied

With respect to the surgical techniques, only complexity grades 1 and 2 breast-conserving procedures were applied according to the classification system of Hoffmann and Wallwiener.^31^ As long as no clinical involvement of the skin (which almost never occurs in these early cases of breast cancer) was suspected, we did not resect skin. To close any defect, we routinely fashioned glandular rotation flaps, mobilizing up to 25 % of the glandular body. For the study cohort, there was no usage of more complex oncoplastic techniques (e.g., pedicle, free flap, fat grafts, implants, or reduction mammoplasty). The surgeon made the decision of how much volume replacement was necessary individually.

### Statistical Analysis

Scores were derived for each of the BREAST-Q’s domains. These were transformed to a scale of 0 to 100 according to the BREAST-Q protocol, with a higher value representing a more favorable outcome. Descriptive statistics included the mean and standard deviation and were used to compare our results with those in former literature. Repeated measures analysis of variance (ANOVA) was used to identify statistically significant differences in the mean satisfaction and well-being levels among the test intervals (baseline [T1], postoperatively [T2], and at the 1-year follow-up evaluation [T3]), with post hoc analysis determining the specifics.

The Greenhouse–Geisser adjustment was used to correct for violations of sphericity. Multiple linear regression analysis was used to identify clinicopathologic variables associated with scores at T3 on the four BREAST-Q scales (“satisfaction with breasts,” “psychosocial well-being,” “physical well-being,” “sexual well-being”). Patients who received a secondary mastectomy between T2 and T3 were excluded from the multiple regression analysis. The regression variables were included in a forward-selection manner because this was an exploratory analysis for risk factors of an unfavorable outcome at T3. Analysis was performed using SPSS, v. 26 (IBM, Chicago, IL, USA).

## Results

### Patient Characteristics

Between July 2017 and May 2018, 259 patients were recruited to participate in this study. Seven patients were excluded because they opted for mastectomy instead of BCT as their surgical procedure, and two patients were unable to complete the questionnaire because of language difficulties. Thus, 250 patients completed the preoperative questionnaire (T1) and received the postoperative module 1 week after surgery (T2). The postoperative questionnaire was returned by 219 (87.6 %) of the 250 patients. The follow-up questionnaire 1 year after surgery (T3) was returned by 188 (75.2 %) of the 250 patients. Seven patients (2.8 %) were censored from the statistical analysis of follow-up data at T3 because they had received a secondary mastectomy during the first year after the index BCT procedure.

The mean age of the cohort was 58 ± 11 years, and the mean BMI was 26.3 ± 5.1 kg/m^2^. Most of the patients had an invasive carcinoma (88.8 %) with a pT1 stage (54.6 %) but no lymph node involvement (66.5 %). Additional patient characteristics can be found in Table 1.

**Table 1:** Patient characteristics at baseline (*n* = 250)

Characteristic	*n*	%
Histology		
*In situ* carcinoma	24	9.6
Invasive carcinoma	223	89.2
Microinvasive carcinoma	2	0.8
Other	1	0.4
Neoadjuvant CHT		
Yes	67	26.8
No	183	73.2
Adjuvant CHT		
Yes	32	12.8
No	218	87.2
Type of surgery		
Lumpectomy	224	89.6
Quadrantectomy	24	9.6
Other	2	0.8
Type of incision		
Radial	46	18.4
Circular	85	34.0
Periareolar	104	41.6
Fishmouth-shaped	13	5.2
Other	2	0.8
Axillary surgery		
SLNE only	176	70.4
ALNE only	46	18.4
SLNE + ALNE	5	2.0
None	23	9.2
(y)pN		
N0	167	66.8
N+	60	23.9
No axillary surgery performed	23	9.2
(y)pT		
T0	24	9.6
Tis/DCIS	31	12.4
T1	137	54.6
T2	55	22.0
T3	1	0.4
T4	1	0.4
Missing data	1	0.4

*CHT* chemotherapy, *SLNE* sentinel lymphadenectomy, *ALNE* axillary lymphadenectomy, *DCIS* ductal carcinoma *in situ*

### BREAST-Q Scores of the BCT Module

In the BREAST-Q, each scale produces an independent score from 0 to 100 and must be interpreted separately because no overall BREAST-Q score is provided. Depending on the research question, researchers and clinicians also can use only parts of the whole questionnaire because the scales are independent of each other. Furthermore, no predefined cutoffs for the score value of each scale, distinguishing between a favorable or unfavorable outcome, are provided.

For “satisfaction with breasts,” the scores were the lowest at baseline (mean, 61 ± 19) and increased over time, as shown by the scores at T2 (mean, 66 ± 18) and T3 (mean, 67 ± 21) (Table 2). A repeated measures ANOVA with a Greenhouse-Geisser correction determined that the mean satisfaction levels showed a statistically significant difference between measurements (F[1.89, 322.6] = 6.01; *p* = 0.003). Bonferroni-adjusted post hoc analysis, presented in Table 3, showed a significant difference in “satisfaction with the breasts” between T1 and T2 (*p* = 0.045), as well as between T1 and T3 (*p* = 0.008).

**Table 2:** Results of the BREAST-Q BCT module

BREAST-Q scale	T1/pre-op		T2/3-4 weeks post-op		T3/1 year post-op	
	*n*	Mean ± SD	*n*	Mean ± SD	*n*	Mean ± SD
Satisfaction with breast	250	61 ± 19	216	66 ± 18	179	67 ± 21
Psychosocial well-being	246	71 ± 16	215	71 ± 18	181	74 ± 19
Physical well-being	247	82 ± 17	199	28 ± 13	179	33 ± 13
Sexual well-being	219	63 ± 17	182	56 ± 19	155	61 ± 23
Satisfaction with information	–	–	199	60 ± 18	–	–
Satisfaction with surgeon	–	–	186	73 ± 23	–	–
Satisfaction with medical team	–	–	213	88 ± 17	–	–
Satisfaction with the office staff	–	–	214	86 ± 18	–	–

*BCT* breast-conserving therapy, *SD* standard deviation

**Table 3:** Bonferroni-adjusted post hoc analysis of the BREAST-Q BCT module

		BREAST-Q scales			
		Satisfaction with breast	Psychosocial well-being	Physical well-being	Sexual well-being
Repeated measures ANOVA with a Greenhouse-Geisser correction					
		F(1.89, 322.64) = 6.01 *p* = 0.003 Partial η^2^ = 0.03^a^	F(1.83, 309.85) = 3.69 *p* = 0.03 Partial η^2^ = 0.02^a^	F(1.73, 264.3) = 1060.34 *p* = 0.00 Partial η^2^ = 0.87^a^	F(2, 286) = 13.04 *p* = 0.00 Partial η^2^ = 0.08^a^
Bonferroni-adjusted post hoc analysis, *p* values					
T1	T2	**0.045**^b^	1.000	**0**.**000**^b^	0.000
	T3	**0.008**^b^	0.067	**0.000**^b^	1.000
T2	T1	**0.045**^b^	1.000	**0.000**^b^	**0.000**^b^
	T3	0.755	0.134	**0.000**^b^	**0.000**^b^

*BCT* breast-conserving therapy, *ANOVA* analysis of variance

^a^Partial η^2^ = effect size, ^b^*p* < 0.05

The mean scores for “psychosocial well-being” were relatively constant, remaining the same between T1 (mean, 71 ± 16) and T2 (mean, 71 ± 18) and increasing slightly at T3 (mean, 74 ± 19) (Table 1). Post hoc analysis did not show any significance in the change over time.

Surprisingly, the “physical well-being” scores of the BCT dropped substantially from the preoperative value (T1: mean, 82 ± 17) to the follow-up value (T2: mean, 28 ± 13). “Physical well-being” increased slightly by T3 (mean, 33 ± 13), without reaching the baseline level. The results for “sexual well-being” showed a significant decrease from T1 (mean, 63 ± 17) to T2 (mean, 56 ± 19), reaching the baseline level at T3 (mean, 63 ± 22).

### Factors Predicting HRQOL After Breast Cancer Treatment

Table 4 shows the factors entered into a multiple linear regression analysis to evaluate predictive factors for the satisfaction and well-being scales one year after treatment. Lower “psychosocial well-being” at baseline (T1), high BMI, and type of incision were predictive factors (*p* < 0.1) for lower “satisfaction with breasts” in the 1-year follow-up (T3) multiple regression analysis.

**Table 4:** Multiple linear regression analyses of the main scales in the BREAST-Q BCT module and clinicopathologic risk factors

	T3: satisfaction with breasts (*n* = 179)		T3: psychosocial well-being (*n* = 181)		T3: physical well-being (*n* = 179)		T3: sexual well-being (*n* = 155)	
	*p* Value	B	*p* Value	B	*p* Value	B	*p* Value	B
Model-adjusted R^2^		0.103		0.300		0.200		0.375
T1: satisfaction with breasts	0.212		0.631		**0.007**^a^	–0.180	0.578	
T1: psychosocial well-being	**0.028**^a^	0.283	**0**.**000**^a^	0.601	**0.003**^a^	0.224	0.978	
T1: physical well-being	0.478		0.355		**0.001**^a^	0.214	**0.008**^a^	0.268
T1: sexual well-being	0.295		0.183		0.582		**0.000**^a^	0.580
Age at surgery (years)	0.867		0.341		0.155		0.367	
BMI at surgery	**0.048**^a^	–0.772	0.869		0.997		0.370	
Type of surgery (reference category, see Table 1)	0.816		0.668		0.502		0.620	
Type of incision (reference category, see Table 1)	**0.092**^a^	–3.968	0.803		0.510		0.454	
Type of axillary surgery (reference category, see Table 1)	0.619		0.201		0.992		**0**.**004**^a^	–9.414
Re-excision	0.789		0.846		0.912		0.452	
Pathologic tumor size (mm)	0.175		0.559		0.885		0.372	
Resected tissue weight (g)	0.649		0.551		0.342		0.391	
pT	0.869		**0.003**^a^	–0.315	**0**.**050**^a^	0.139	0.387	
pN	0.374		0.763		0.499		**0.017**^a^	1.374
Neoadjuvant CHT	0.955		0.886		0.802		0.895	
Adjuvant CHT	0.631		**0.014**^a^	10.039	0.112		**0.090**^a^	6.953
Endocrine therapy	0.876		0.762		0.167		0.824	
Seroma	0.761		0.120		**0.074**^a^	5.821	**0.072**^a^	9.466
Impaired wound healing	0.373		0.752		0.294		0.208	

*BCT* breast-conserving therapy, *B* regression coefficient, *Model-adjusted R2*, adjusted coefficient of determination, *BMI* body mass index, *CHT* chemotherapy

^a^*p* < 0.10

## Discussion

Although analysis of postoperative PROMs is continuously finding its way into research studies and outcome evaluation, surveys including evaluation of preoperative or pretreatment data still are rare.^27^ Our study investigated the impact of breast-conserving surgery on patients’ QoL and found a significant improvement in patients’ HRQoL over time on three of the four scales. Because longitudinal studies using the BREAST-Q are rare, studies to which ours can be compared are sparse. Our results are mostly consistent with those of a prospectively followed cohort from Poland by Krzos et al.,^32^ showing that most long-term survivors of breast cancer ultimately reach QoL levels comparable with the baseline level.

In our study, the mean “satisfaction with breasts” scores were slightly higher than those of Krzos et al.^32^ The reported mean scores were 56.0 before surgery, 63.0 at 3 months after surgery, and 62.2 at the 1-year follow-up evaluation.^30^ In another study by O’Connell et al.^30^ evaluating 200 patients 1 to 6 years after BCT, the mean “satisfaction with breasts” score was 69.9, comparable with our results at the 1-year follow-up evaluation. Similarly, Lagendijk et al.^27^ reported a mean “satisfaction with breasts” score of 65.7 in a cohort of 257 patients. Dolen et al.^33^ reported findings with slightly higher mean scores of 61.9 before and 74.2 at 6 months after radiotherapy.

Although the increase in the scores for “satisfaction with breasts” over time was only slight in our study, this increase was statistically significant for both T2 and T3 compared with T1, whereas the increase between T2 and T3 showed no significant difference. Contrary to our initial expectations, the mean “satisfaction with breasts” score was higher immediately after surgery than before.

In a prospective cohort study with 849 patients using the Breast Cancer Treatment Outcome Scale (BCTOS) questionnaire, the aesthetic status decreased from before surgery to shortly after surgery and to the long-term follow-up evaluation.^34^ The “aesthetic status” of the BCTOS comprises items such as “breast shape,” “nipple appearance,” and “scar tissue.”

The main outcome measurement criterion of the BCTOS is the patients’ assessment of the treated breast compared with the untreated breast, whereas the BREAST-Q explores “satisfaction with the breasts” in a more holistic way. Our findings suggest that the “satisfaction with breasts” scale of the BREAST-Q measures different aspects of surgical outcome and HRQOL than the BCTOS, with less focus on the aesthetic outcome.

A possible interpretation of our finding is that the patient’s knowledge of a tumor inside her breast may lead to lower satisfaction. Thus, the satisfaction could increase after tumor removal despite an aesthetic impairment caused by the surgical procedure. Although aesthetic status is definitely part of QoL, assessment of satisfaction is superior because patients might accept worse aesthetics in exchange for tumor-free survival, being satisfied with the outcome.

In our study, BMI, preoperative psychosocial well-being, and type of incision appeared to be significant predictors of lower satisfaction with breasts at the follow-up evaluation. The significance of type of incision is especially interesting because it represents the relation of surgical techniques and patient satisfaction, which has already been reported in various studies.^34^–^36^ The study of O’Connell et al.^30^ and other studies^37^,^38^ also have identified BMI as a risk factor, but otherwise, our results in this regression analysis are not consistent with those of other studies because impaired wound healing and axillary surgery were no predictors in our analysis. This might have been due to the short time frame of 1 year for our study because the time from surgery in O’Connell et al.^30^ ranged from 1 to 6 years. Axillary surgery might gain a more important role with a longer time frame because lymph edema may develop later after the surgical intervention and radiation.^39^

The results of the “psychosocial well-being” scales were similar at all three measurement times, without significant changes over time. This finding was consistent with the theory that psychological dissatisfaction goes deeper than the current situation and partially depends on personality traits,^40^ which would have already been present in the preoperative inquiry. Studies and meta-analyses evaluating psychological well-being among patients have found that it can be improved with behavioral interventions^41^ or by successful change of personality traits.^42^

In our study, tumor stage was a significant predictor of psychosocial well-being. This was consistent with findings stating that receiving the diagnosis of an early stage of breast cancer already has an impact on the mental domains of HRQOL.^43^ Furthermore, our analysis showed that psychosocial well-being before treatment was a significant predictor of psychosocial well-being in the follow-up evaluation. This confirms the recommendations of targeted psychological interventions accompanying breast cancer therapy for high-risk groups, which are identified before treatment to prevent dissatisfaction, low psychosocial well-being, and consecutive depression.

The results of our study may indicate that more attention and effort should be dedicated to the long-term “psychosocial well-being” of the patients because no improvement in that scale was observed in the follow-up evaluation, although all patients were offered psycho-oncological attendance during therapy. Easy-to-implement psychological interventions have been developed and evaluated in randomized-controlled trials, with many showing positive results.^44^,^45^

The potential for developing more targeted, quantifiable interventions to be broadly disseminated is substantial.^44^,^46^ Such interventions not only would improve psychosocial well-being but also may have the potential to promote and maintain physical health.

Our results showed the greatest changes over time for “physical well-being.” Although physical well-being seems to be sufficiently high before surgery (mean, 82 ± 17), a considerable decrease occurs after surgery (mean, 28 ± 13), with a slight increase in the 1-year follow-up evaluation (mean, 33 ± 13), without reaching baseline levels. All differences were statistically significant, showing that distinct improvement in the physical level occurs during the first year after surgery. These results do not match our expectations, and means of “physical well-being” scores reported by other studies after BCT have been noticeably higher (e.g., 67 during a follow-up period of 12 months,^32^ 78 during a median follow-up period of 28 months,^47^ 80 during 5 to 7 years from surgery to survey,^26^ and 81 during a median follow-up period of 5.5 years.^28^ These studies all had different time frames for the follow-up evaluation, which surely in part explains the variation in the results.

The physical well-being scores of mastectomy patients often are higher than those in our results. Howes et al.^48^ compared the BREAST-Q results of patients who received BCT, mastectomy, and mastectomy with reconstruction. The BCT cohort had the lowest physical well-being scores, but they still were almost double the scores in our cohort. Only a study by Dolen et al.^33^ showed similar findings with “physical well-being of chest” scores of 49.4 in a 6-month follow-up evaluation.

All studies seem to show a general tendency of improvement over time. Although the survey by Krzos et al.^32^ had only a 12-month follow-up period, their mean “physical well-being” score still showed a large difference to the score in our study. A noticeable difference was that the percentage of women receiving axillary lymph node dissection (ALND) in our study (20.4 %) was higher than in the cohort of Krzos et al. 32 (12.1 %), indicating that axillary surgery might have a considerable influence on patients’ physical well-being in the long term. Furthermore, confounding factors could have influenced the physical well-being in our rather heterogeneous cohort (e.g., women who received adjuvant chemotherapy or older women with more comorbidities).

More longitudinal data are needed to determine at what level the physical improvement stops and whether the baseline levels are reached again. Our data confirm that the patients need to be thoroughly advised about all possible treatment options and that BCT, although often possible, might not be the best option for all patients regarding long-term physical well-being.

The “sexual well-being” scale had the lowest response rates at all three assessment times. In our study, the mean “sexual well-being” score decreased after surgery, with a significant difference from the baseline status. At the 1-year postoperative follow-up evaluation, the mean sexual well-being score increased to the baseline level again. This is consistent with the findings from a prospective study of women one year after BCT, which found no significant difference in female sexual dysfunction compared with a healthy control group.^49^ Another study evaluating breast-specific sensitivity and sexual function with regard to surgical method reached the same conclusion.^50^

The scales concerning satisfaction with care were administered only once, 3 to 4 weeks after surgery in our study. The “satisfaction with information” scale had the lowest score (mean, 70 ± 18), followed by “satisfaction with surgeon” (mean, 73 ± 23). Other studies show the same ranking order of the scales concerning the experience of care but higher mean scores.^28^,^30^ The timing of administration could be important for these scales.^51^ If the scales are administered too late, the results may be confounded by the overall satisfaction with the therapy result. If administration is too early, patients might not yet be able to review the different parts of care separately because they would still be suffering from side effects of the surgery.

Clinicians might consider administering the “satisfaction with information” scale before surgery as well as evaluating whether the patients feel sufficiently informed about the upcoming procedure. This might be especially interesting with regard to the association of positive outcomes with patient involvement and shared decision-making in the management of breast cancer.^52^–^54^ Administration of these scales years after treatment might be useful only to a limited extent because the scales are composed of detailed questions for which the answers might not be as memorable after a long time. Furthermore, the “satisfaction with surgeon” scale will be of advantage only in hospital systems wherein the patients have some quality interaction with their surgeon outside of surgery, and the German university hospital system does not always meet this requirement. Thus, some adaptation of that scale might be needed in case of irregular results or low response rates.

As one of its strengths, this study was one of very few longitudinal publications of the BREAST-Q BCT module including a baseline dataset and reporting on a large cohort of women. Our baseline data could be used as a benchmark for further studies until studies with a larger patient cohort are recruited (e.g., the COSMAM trial^55^).

Among its limitations, the study was restricted to a single center with a rather homogeneous patient collective and the limited time frame. Further longitudinal research is necessary to assess the long-term evolution of patient satisfaction and well-being because breast cancer survivors have an ongoing need to process the life-changing effects of cancer.^56^

Because no recommendations for interpreting the results of the BREAST-Q exist to date, it is difficult to deduce clinical consequences from seemingly small differences in results (e.g., 5 points on the 100-point scale). As soon as sufficient benchmark data are reported in literature, the clinical meaning might become more easily deductible.

## Conclusion

Whereas one year after surgery, breast-cancer patients treated with BCT are as psychosocially and sexually satisfied with their breasts as before treatment, the physical well-being level will not yet have reached baseline levels. Our results indicate a large need for further longitudinal research to evaluate the HRQOL of breast cancer patients with a standardized PROM that includes preoperative baseline data and follow-up assessments. The BREAST-Q can serve as a possible standard PROM in clinical practice for quality assessment and in clinical research trials because it is able to identify even slight alterations in patient satisfaction over time. However, a need still exists for more benchmark data to derive clinical consequences from BREAST-Q results.

In this study, psychosocial well-being, BMI, and type of incision were significant predictors of satisfaction with breasts, which shows that both the aesthetic and psychological aspects are important with regard to the outcomes of breast cancer surgery. High-level standardization of surgical techniques and patient-reported outcomes should be realized in subsequent prospective studies to improve outcome from the patient’s perspective. Further research with controlled clinical trials is necessary to determine the specifics of the influence that different surgical incision methods have on patient satisfaction.
